# Forage quality and fermentation dynamics of silages of Italian ryegrass (*Lolium multiflorum* Lam.) wilted for varying periods

**DOI:** 10.5713/ab.24.0251

**Published:** 2024-06-25

**Authors:** Yan Fen Li, Li Li Wang, Young Sang Yu, Xaysana Panyavong, Li Zhuang Wu, Jong Geun Kim

**Affiliations:** 1 Graduate School of International Agricultural Technology, Seoul National University, Pyeongchang 25354, Korea; 2 Research Institute of Eco-friendly Livestock Science, Institute of GreenBio Science Technology, Seoul National University, Pyeongchang 25354, Korea

**Keywords:** Fermentation, Forage Quality, Italian Ryegrass Silage, Wilting Time

## Abstract

**Objective:**

This trial was conducted to explore the impact of different wilting time of Italian ryegrass (IRG) in the field on the nutritional quality and fermentation characteristics of its silage.

**Methods:**

The harvested IRG was directly wilted in the field for 0 day (W0), 1 day (W1), 2 days (W2), and 3 days (W3), respectively, and tedded every 6 hours. And the preserved IRG was sampled at 1, 2, 3, 5, 10, 20, 30, and 45 days after ensiling and three replicates per treatment.

**Results:**

With the extension of wilting, the dry matter (DM) content and pH value of wilted IRG gradually increased (p<0.05). There was a downward trend in; neutral detergent fiber (NDF), acid detergent fiber (ADF), and hemicellulose with the increase of wilting time, but only W2 and W3 were significantly different from W0 (p<0.05). Crude protein (CP), *in vitro* DM digestibility), total digestible nutrients (TDN), and relative feed value decreased significantly with the increase of wilting time (p<0.05), except for W1. After 45 days of ensiling, W1 had the highest CP, TDN, and the lowest ADF and NDF. During ensiling, the increase of acetic acid and the decrease of WSC in W0 and W1 were similar, but the accumulation rate of lactic acid in W0 was faster than that in W1, resulting in the lowest pH value in W0. After 5 days of ensiling, the ratio of lactic acid to acetic acid in W1 stabilized at around 3:1, while W0 kept changing.

**Conclusion:**

Italian ryegrass that wilted in the field for 1 day effectively improved the dynamic changes in CP, TDN, ADF, and NDF and fermentation quality of silage. Therefore, in practice, W1 was more recommended in production of IRG silage.

## INTRODUCTION

Italian ryegrass (*Lolium multiflorum* Lam., IRG) is considered an important seasonal forage for the maintenance of ruminants due to its high nutritional qualities and is widely cultivated worldwide, such as in temperate, subtropical and tropical plateau regions [1], including Korea. Recently, in Korea, the utilization of IRG has been well confirmed and the cultivated area has been gradually increased [2]. Livestock farms have increasingly preferred IRG, which has superior silage quality and palatability, over existing forages such as rye and barley, making it one of the winter forage crops in Korea. In 2018, for winter forage crops, the area of IRG cultivation was 169,000 hectares, and the remaining 6,000 hectares were planted with winter crops such as rye and barley, of which IRG accounted for more than 96.6% [3]. Unfortunately, the best time to harvest IRG is the rainy season, so its storage is an enormous challenge for farmers. The hay making often fails due to rain, silage becomes the best option.

At present, ensiling has become a common method of forage preservation. High-quality silage can effectively maintain nutrients and palatability of forage. It mainly relies on the fermentation of lactic acid bacteria (LAB) to ferment the carbohydrates in fresh forage into organic acids mainly lactic acid, lower the pH value, and limit the activities of undesirable bacteria [4–6]. Ensiling is an ideal method for maintaining sufficient forage, as it serves as a significant component in the ration of ruminants, not only to effectively alleviate the winter feed shortage problem, but also to help overcome the seasonal imbalance between livestock feed consumption and available forage throughout the year [6,7].

Italian ryegrass silage has become an indispensable source of forage for farms in Korea. However, it is difficult to naturally ferment high-moisture forage to produce high-quality silage. During the ensiling, high-moisture not only causes a large loss of nutrients, but even results in the feed to deteriorate and produce toxic substances, which eventually not only damages the animals’ health but also leads to irreparable and significant economic losses to the farm [4,8]. Field wilting prior to ensiling has been widely performed as it is an effective method of reducing moisture content and enhancing silage characteristics and avoiding silo seepage losses [4]. Achieving rapid field wilting is crucial for avoiding the loss of dry matter (DM) and nutritional value. However, the degree of moisture loss and wilting conditions vary, depending on weather conditions, especially temperature, solar radiation, wind conditions, and crop characteristics [9]. If the harvested forage is immediately conditioned and spread in the field, the drying rate can be accelerated and the loss of DM and nutrient can be reduced [4].

Liu et al [10] concluded that wilting before ensiling reduces the content of acetic acid and ammonia nitrogen and improves the fermentation quality, especially heavy wilting limits the production of ammonia nitrogen (NH_3_-N) in stylo silage to a greater extent. Many studies have shown that wilting can effectively inhibit the growth of undesirable microorganisms to reduce nutrient loss [4,10,11]. However, some chemical changes may occur during the field wilting process, resulting in nutrient loss, such as the reduction of carbohydrates, protein decomposition, and changes in microbial communities. The current researches have mainly explored factors that affect the wilting speed and quality, and the impact of wilting or not on silage quality, but there are no more references on the influence of wilting time on ensiling dynamics in nutrition value and fermentation profiles of IRG. Thus, this experiment aimed to compare the effects of different wilting time on the forage quality and fermentation dynamics of IRG silage.

## MATERIALS AND METHODS

### Forage cultivation and silage making

**Figure 1:**
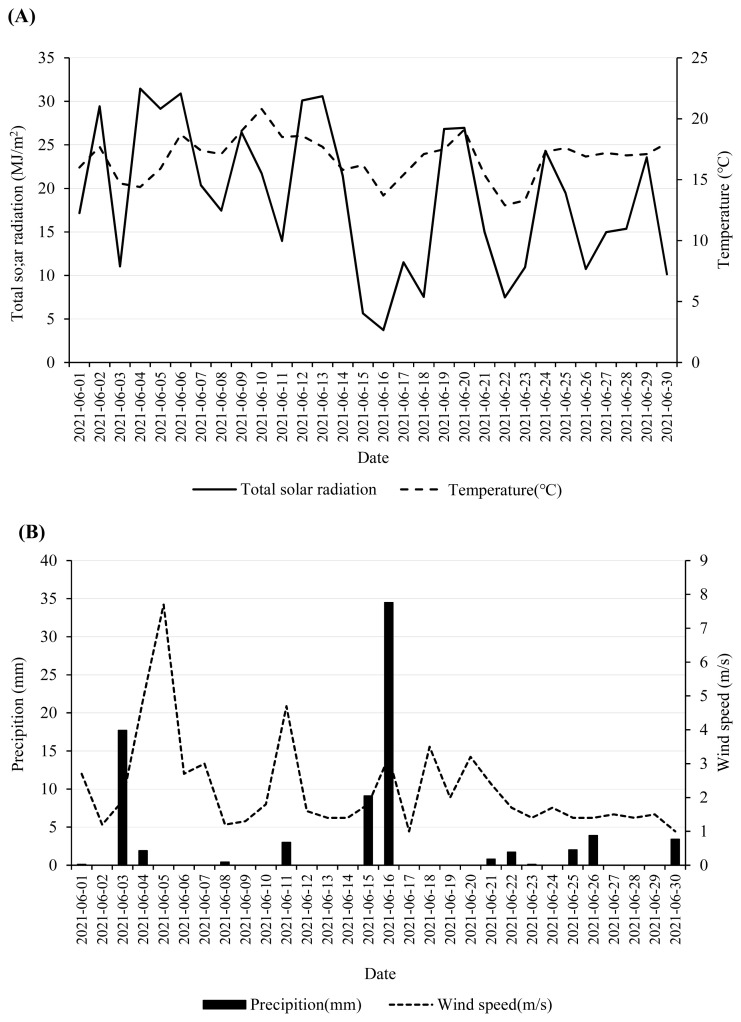
Average temperature and total solar radiation (A), wind speed and precipitation (B) during the experimental month (June 1 to 30, 2021). Source: Korean Meteorological Administration.

The IRG was harvested on June 6, 2021, in the experimental field of Pyeongchang Campus, Seoul National University (37°32′46.1″ N, 128°26′17.9″ E) in Republic of Korea. The temperature, wind speed and rainfall during the experimental month are presented in Figure 1. The meteorological information is registered as mean temperature 17.73°C, average precipitation 0.13 mm, average wind speed 2.30 m/s, average humidity 75.03% during wilting period (June 6 to 8, 2021). The harvested IRG was directly wilted in the field for 0, 1, 2, and 3 days, respectively, and tedded 4 times every day. Italian ryegrass treatments were as follows: 0-day wilting (W0), 1-day wilting (W1), 2-day wilting (W2), and 3-day wilting (W3). Wilted IRG was chopped into 2 to 3 cm lengths and mixed thoroughly for each treatment. A 400-grams wilted IRG were packed into vacuum polyethylene plastic bags (Food grade, 28 cm×36 cm, Korea) and immediately sealed using a vacuum packer (FM-06; Aostar, Seoul, Korea). The IRG stored at ambient temperature (24°C to 30°C) was sampled at 1, 2, 3, 5, 10, 20, 30 or 45 days after ensiling.

### Analytical procedures

About 150-grams fresh material and silage were placed in air - forced drying oven at 65°C for 72 hours to analyze DM. The dried samples were ground into 1-mm particle size using a mill (Thomas Scientific, Swedesboro, NJ, USA) for nutrients analysis. Total nitrogen was determined by an elemental analyzer (Euro Vector EA3000; EVISA Co., Ltd, Milan, Italy) according to Dumas method [12]. The water-soluble carbohydrates (WSC) was analyzed by the anthrone method [13]. Neutral detergent fiber (NDF) and acid detergent fiber (ADF) were performed via the method of Van Soest et al [14] using an Ankom2000 fiber analyzer (Ankom Technology Corp., Fairport, NY, USA) and sodium sulfite and α-amylase were added for NDF producer. *In vitro* DM digestibility (IVDMD) was quantified after the 48-h incubation by an Ankom DaisyII incubator (Ankom Technologies, Inc., USA), according to the method of Goering and Van Soest [15]. The rumen donor (Holstein) and rumen fluid preparation were described as reported by Ahmadi et al [16]. Total digestible nutrients were got by the equation (88.9 − [0.79× ADF %]) [17]. Relative feed value (RFV) was calculated by the following formula described by Rohweder et al [18]. Digestibility of dry matter (DDM) = 88.9–(0.779×ADF%); dry matter intake (DMI) = 120/NDF%; RFV = (DMI×DDM)/1.29.

Forage extract was prepared for the analysis of fermentation profile followed by Wei et al [19]. The pH value of extract was determined immediately by the AB 150 pH meter (Fisher Scientific International, Inc., Pittsburgh, PA, USA). Organic acids were determined by HPLC system (Detector, RI; Column, Agilent Hi-Plex H; Agilent Technologies 1260 Infinity, Santa Clara, CA, USA) in accordance with the previously prescribed procedures [19]. NH_3_-N was measured by a UVIDEC-610 spectrophotometer (Jasco, Tokyo, Japan) [20].

### Statistical analysis

Data were analyzed by two-way analysis of variance with general linear model Proc in SPSS (IBM Corp. Released 2016. IBM SPSS Statistics for Windows, Version 24.0.; IBM Corp., Armonk, NY, USA). The analysis model was expressed as: Y_ij_ = μ+T_i_+D_j_+(T×D)_ij_+ɛ_ij_, where Y_ij_ = observation, μ = mean, T_i_ = effect of treatments (wilting time), D_j_ = day of ensiling, (T×D)_ij_ = interaction effect of treatment × day of ensiling, and ɛ_ij_ = error term. Each silage bag was considered the experimental unit in the model. Duncan’s multiple range test was used to assess the significance of differences between treatment groups. If p<0.05, there was a significant difference.

## RESULTS AND DISCUSSION

### Chemical composition prior to ensiling

The moisture of the forage plays an important role in silage fermentation quality. Alcoholic fermentation may occur if low-moisture silage is prepared with a high-sugar grass [21] and the high moisture content of tropical grasses induces acetic acid fermentation to dominate the silage process [22]. Table 1 shows that there was a positive correlation between DM content and wilting time. With the increase of wilting days, the DM content increased and there was a rapid rise in DM during the first 2 days of wilting. Our result was consistent with Ribas et al [23]. Furthermore, Polak and Jančová [24] wilted grass and mixed forage crops for 2 days and measured the DM content in the morning and afternoon each day and observed that the DM increased as the wilting time increased, and there are disadvantages to excessive wilting. This may be because longer wilting time can result in reduced population of LAB and the proliferation of undesirable bacteria. Pahlow et al [25] indicated that prolonged exposure of forage to sunlight can negatively affect silage quality. Similarly, in our study, it can be found there was an increase in NDF, ADF, and hemicellulose (HEM) and decline in IVDMD before ensiling. The effect of wilting time on digestibility was opposite to that of NDF and ADF, W0 was the highest. This is similar to Keles et al [26], who demonstrated wilting for 72 hours leaded to lower IVDMD compared with 24 or 48 hours. Prolonged wilting can lead to increased microbial activity, which may contribute to further breakdown of soluble components and an increase in fiber content. In addition, wilting results in a concentration of the plant’s cell wall components. There was a decline in CP as wilting time increased and the CP dropped significantly after 24 hours. Likewise, Müller et al [27] showed that the CP content of three different fodder crops decreased with increasing wilting time. Furthermore, studies have proven that CP in forage decreased with increasing wilting time or DM content [23,27]. The decrease in protein content may be due to soluble proteins in the plant material leaching out with water as the forage dries during the wilting process. In a previous study, although different, the pH of all three forage crops increased to 5.48–6.84 as the amount of DM increased [27]. Similarly, the pH value gradually changed from 5.86 to 6.41 as the DM content of IRG increased from 30.61% to 75.66% during wilting in our experiment. This may be due to the proteolysis, the activities of aerobic microbes or the respiration of plant cells during wilting. A similar result where the pH of clover increased with prolonging wilting time was also reported by Orosz [28]. It was clarified that the DM content was considered to vary in parallel with the pH value [29].

### The effects of wilting time and ensiling days on the chemical compositions of IRG silage after ensiling

During ensiling, the DM content had similar changing trends for W0, W1, W2, and W3 (Table 2). There was a decrease in DM with increasing ensiling time (p<0.05) but W0 and W1 had a larger drop relative to W2 and W3. This may be attributed to the moisture content at the initial of the IRG silage. The moisture of the forage is an important factor affecting the fermentation quality of silage. Wilting time significantly affected the CP content (p<0.01) but the ensiling days had no significant effect on CP (p>0.05) (Table 2). There were ups and downs in CP content of W0 and W1 changes, but W2 and W3 were almost maintained in a stable state throughout the process, which may be related to the moisture content. This is because water activity affects microbial growth and enzyme activity [30]. Overall, the CP was increased in W1, it may be inferred that special microbial activities synthesize amino acids or peptides into proteins, or dead bacteria are degraded into protein.

During ensiling, there is no significant change in NDF and ADF of W0, W1, W2, and W3 (p>0.05), among which W1 and W2 had frequent changes and in a wide range (Table 2). The occurrence of this phenomenon may be due to the metabolic activity of microorganisms during ensiling for W1, and may be related to the changes in DM content for W2. W3 and W2 had the higher NDF, ADF, and HEM compared to W0 and W1 (p<0.01) during ensiling. And ADF was the lowest in W1 (p<0.01). This could be explained as the cell wall is degraded by plant enzymes, cellulolytic microorganisms or acid hydrolysis. The higher NDF and ADF concentration were reflected in a lower TDN and RFV for W2 and W3. This is line in with the theory that RFV and TDN are calculated based on NDF and ADF [18]. TDN was relatively stable overall in W0, W1, and W3, but W2 changed significantly. W1 had the highest TDN, followed by W0 (Table 3). British turf with wilting for 24, 48, and 72 hours was ensiled for 295 days, which proved that wilting time had a significant effect on DM and IVDMD [26]. In the experiment, it was reported that IVDMD increased and decreased during the ensiling.

### Fermentation quality after ensiling

**Figure 2:**
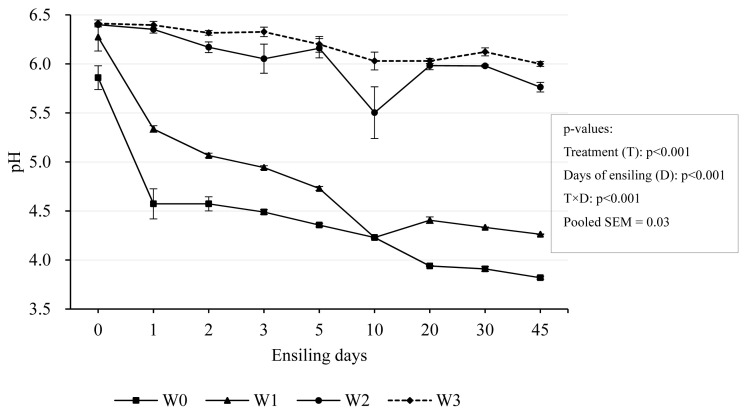
Dynamic changes of pH during ensiling. Treatments were 0-day wilting (W0), 1-day wilting (W1), 2-day wilting (W2), 3-day wilting (W3). T×D, the interaction of treatments and ensiling days. SEM, standard error of mean. If p<0.05/0.001, there was a significant difference.

The effects of the wilting time and days of ensiling on silage pH are illustrated in Figure 2. During the first 10 days of ensiling, the pH had the greatest drop in W0 and W1, but little change in W2 and W3. The rapid drop in pH value can effectively reduce proteolysis, inhibit undesirable microorganisms and reduce nutrient loss [31,32]. The pH value in W0 and W1 dropped to 4.23 on the 10th day of ensiling while W2 and W3 have a higher pH value, 5.50 and 6.03 respectively. It is likely deduced that as forage wilts and loses moisture, the concentration of these buffering agents increases, making it harder for the pH to decrease rapidly once fermentation begins. After that only W0 had the further declines and reached 3.81 after 60 days-ensiling. Studies indicated that a pH between 3.5 and 4.5 indicates a successful fermentation for forage silage [32]. It suggested that W0 and W1 were successfully fermented while there was a poor fermentation in W2 and W3. Furthermore, studies reported that high DM content in forage do not contribute to decreasing silage pH levels [33]. Therefore, our experimental results were further confirmed.

**Figure 3:**
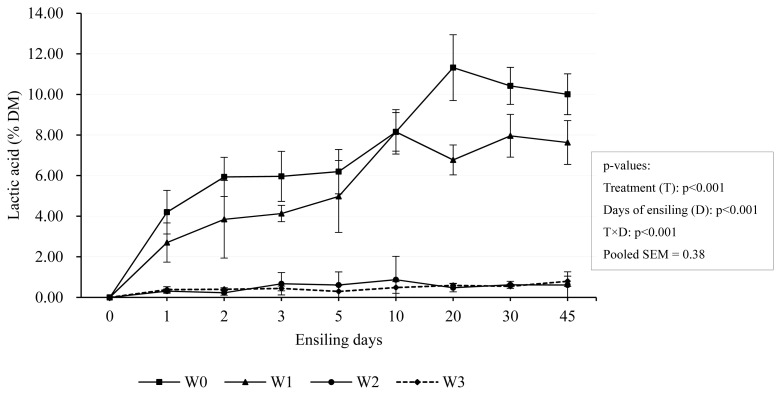
Dynamic changes of lactic acid concentration during ensiling. Treatments were 0-day wilting (W0), 1-day wilting (W1), 2-day wilting (W2), 3-day wilting (W3). T×D, the interaction of treatments and ensiling days. SEM, standard error of mean. If p<0.05/0.001, there was a significant difference.

The change in pH value is closely related to the lactic acid concentration, and the rapid decrease in pH value is attributed to the low pKa of lactic acid [34]. Our experimental result likewise indicated this. It was reflected in changes in lactic acid concentration during ensiling. Lactic acid concentrations in both W0 and W1 increased rapidly and were consistent at day 10 (8.16% DM), while there was still a rapid increase in W0 afterward but stabilized in W1 (p<0.001) (Figure 3). This coincided with the decrease in pH. However, the lactic acid concentration of W2 and W3 was quite low, lower than 1% in DM. The lactic acid concentration in IRG silage exceeded the typical range (20 to 40 g/kg DM) in this experiment, which may be attributed to the higher moisture content (600 to 700 g/kg) of W0 and W1 during ensiling, which promoted the formation of a large amount of lactic acid Kung Jr et al [34]. Wilting level had significant effects on IRG fermentation by limiting microflora activity, as shown by a decrease in fermentation product with increasing DM [35]. In the study, this was reflected in the higher pH (p< 0.001) and lower (p<0.01) lactic acid concentration in the more wilted silages (W2 and W3). Light wilted silage (W1) showed more intense fermentation with higher lactic acid and acetic acid content.

**Figure 4:**
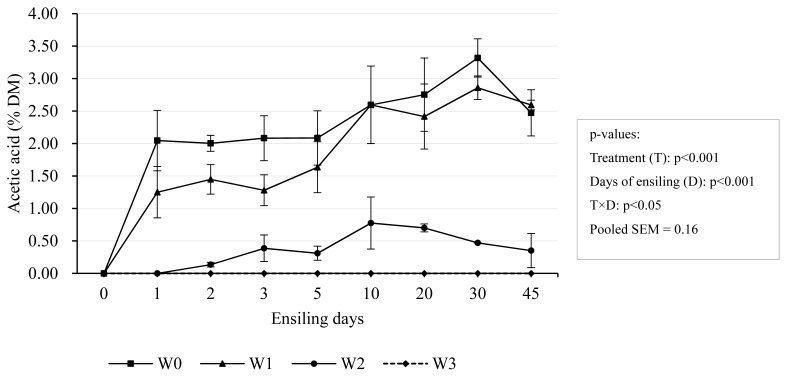
Dynamic changes of acetic acid concentration during ensiling. Treatments were 0-day wilting (W0), 1-day wilting (W1), 2-day wilting (W2), 3-day wilting (W3). T×D, the interaction of treatments and ensiling days. SEM, standard error of mean. If p<0.05/0.001, there was a significant difference.

The acetic acid content in W0 and W1 had the largest increase on the first day but then increased slowly while acetic acid was almost not detected in W3 (0.06% DM) (Figure 4). After 45 days-ensiling, W0 and W1 had the highest acetic acid concentration of 2.47% and 2.59% in DM respectively. Acetic acid plays a vital role in silage. The appropriate amount of acetic acid content in silage can not only inhibit yeast and improve the aerobic stability of silage, but can also be absorbed by the rumen and used for energy or incorporated into milk or body fat. Thus, less is not always better, acetic acid usually accounts for 1% to 3% DM in silage [34]. The acetic acid content in this experiment was just within this range.

**Figure 5:**
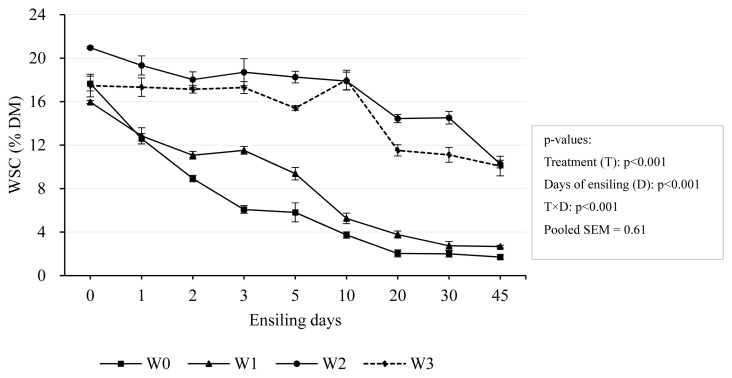
Dynamic changes of WSC concentration during ensiling. Treatments were 0-day wilting (W0), 1-day wilting (W1), 2-day wilting (W2), 3-day wilting (W3). T×D, the interaction of treatments and ensiling days. SEM, standard error of mean. If p<0.05/0.001, there was a significant difference.

The effects of wilting time and days of ensiling on WSC of the IRG are presented in Figures 5. There was gradual decrease in WSC concentration in all silages, and the decline was relatively rapid during the first 5 days of fermentation, followed by a slower decline. W0 had the largest decrease in WSC followed by W1, W2, and W3 (p<0.001). After 45 days-ensiling, WSC consumption rates varied, with W0 reaching the maximum of 90.27%, followed by W1 at 83.27%, then W2 at 51.96%, and finally W3 at 42.39%. This corresponded to the level of wilting. As the wilting time increased, the WSC consumption rate decreased. This may be because moisture content affects bacterial growth and thus prevents WSC from being utilized. Depletion of WSC during ensiling is generally due to the consumption of LAB, converting it into organic acids (mainly lactic acid) [32]. Alternatively, WSC may also be consumed by undesirable bacteria and LAB, with the net result of a low lactic acid level that may not be sufficient to acidify the silages strongly and thus retard the growth of undesirable bacteria [32]. In our experiments, WSC concentration was negatively correlated with lactic acid and acetic acid but positively correlated with pH value. This further showed that the wilting time was prolonged and the moisture content was reduced, which affected microbial activity and inhibited fermentation. This is line in with Valente et al [35].

**Figure 6:**
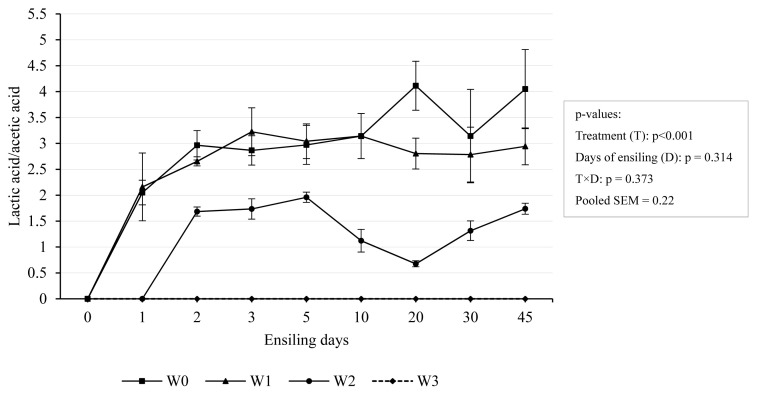
Dynamic changes of lactic: acetic acid ratio concentration during ensiling. Treatments were 0-day wilting (W0), 1-day wilting (W1), 2-day wilting (W2), 3-day wilting (W3). T, treatments; D, the days of ensiling; T×D, the interaction of treatments and ensiling days. SEM, standard error of mean. If p<0.05/0.001, there was a significant difference.

W0 and W1 showed an increase in the lactic acid/acetic acid ratio during the early phase of ensiling, after that W1 tended to be stable but W0 had an increase and drops (Figure 6). After 5 days of ensiling, the lactic acid to acetic acid value of W1 stabilized at around 3:1, while that of W0 stabilized at around 3:1 within 3–10 days and then rose to 4.05:1, and dropped again. It showed that W1 fermentation was basically stable after 5 days of ensiling, while W0 was less stable. W2 had a slower and lower increase and a decrease after 5 days of ensiling, and the maximum value didn’t reach 2:1. The ratio of lactic acid to acetic acid is considered an indicator of the type of fermentation. Researches showed that good silage fermentation usually has a ratio of 2.5 to 3.0 [34]. Therefore, it’s proved that W1 had good fermentation. Silage with a higher than normal ratio of lactic acid: acetic acid may indicate that it is more unstable because acetic acid at lower concentrations is not sufficient to inhibit lactate-assimilating yeasts. In our study, W0 had a lactic acid: acetic acid ratio higher than 3, which may be attributed to W0 having a higher moisture content relative to the other treatments. It also indicated that W0 silage was more unstable. W3 has an abnormal lactic acid: acetic acid ratio, which may be due to low moisture content causing abnormal fermentation. This is line with Kung Jr et al [34], who reported that a lactic acid:acetic acid ratio below 1:1 usually indicates an abnormal fermentation.

## CONCLUSION

In our study, wilted 1-day-wlited IRG silage had greater potential for nutritional value and fermentation profile. There was a significant decline in moisture content, an increase in CP content and a decrease in fiber content (ADF, NDF, and HEM), thereby improving TDN and RFV for 1-day wilted IRG. What’s more, the nutritional value of forage that has wilted for 1 day was effectively retained after ensiling, avoiding nutritional losses caused by excessive moisture before ensiling. Moreover, wilted 1-day-wlited IRG silage was more in line with the needs of livestock farmer for forages. Not only did the its acetic acid content fall within the recommended range, but it was mainly homo-fermented by LAB. Above all, secondary fermentation was less likely to occur compared to direct ensiled feed that did not wilt. Hence, in semblable climates, 1-day wilting is recommended as the optimal wilting time for IRG before ensiling to optimize the nutritional value and fermentation characteristics of IRG silage in our experiment.

## Figures and Tables

**Table 1:** The chemical composition of Italian ryegrass with different wilting time prior to ensiling

Treatments^1)^	DM (% FM)	pH	% DM						RFV	TDN (%)

			WSC	CP	NDF	ADF	HEM	IVDMD		
W0	30.61^d^	5.86^b^	17.67	6.36^a^	66.06^b^	40.28^c^	25.78^ab^	65.87^a^	81.01^a^	57.08^a^
W1	42.78^c^	6.27^a^	15.96	6.31^a^	66.75^b^	41.39^bc^	25.37^b^	61.69^ab^	78.96^a^	56.20^ab^
W2	71.01^b^	6.40^a^	18.96	5.06^b^	70.17^a^	43.27^a^	26.90^a^	58.84^b^	73.17^b^	54.72^c^
W3	75.66^a^	6.41^a^	17.48	4.19^b^	69.41^a^	42.43^ab^	26.99^a^	59.28^b^	74.89^b^	55.38^bc^
Mean	56.43	6.24	18.02	5.48	68.10	41.84	26.26	61.42	77.00	55.85
SEM	0.64	0.13	0.89	0.45	0.72	0.60	0.61	2.36	1.25	0.47

DM, dry matter; FM, fresh weight; WSC, water-soluble carbohydrates; CP, crude protein; NDF, neutral detergent fiber; ADF, acid detergent fiber; HEM, hemicellulose; IVDMD, in vitro dry matter digestibility; RFV, relative feed value; TDN, total digestible nutrients; SEM, standard error of mean.

1) Treatments were 0-day wilting (W0), 1-day wilting (W^1)^, 2-day wilting (W2), 3-day wilting (W3).

a–d Withina column means with different superscripts differ (p<0.05).

**Table 2:** Effect of different wilting period on the dynamic change of DM, CP, NDF, ADF and hemicellulose content of Italian ryegrass silage

Items	Treatments^1)^	Ensiling days (d)								Mean	SEM	p-value^2)^		
	
		1	2	3	5	10	20	30	45			T	D	T×D
DM (% FM)	W0	28.72	28.47	26.50	27.34	27.37	27.93	27.55	27.89	27.72	1.15	<0.001	<0.001	<0.001
	W1	39.40	39.55	39.75	39.95	37.37	39.90	38.99	39.04	38.00				
	W2	71.00	68.53	66.04	70.06	67.41	68.81	69.60	69.28	68.84				
	W3	72.83	73.72	73.54	74.58	72.98	73.24	73.33	75.13	73.67				
	Mean	52.99	52.57	51.46	52.98	48.78	52.47	52.37	52.84	52.06				
CP (% DM)	W0	6.32	7.15	6.90	6.62	6.36	6.03	6.56	6.23	6.52	0.29	<0.001	0.127	0.120
	W1	6.98	7.00	6.66	6.60	6.36	6.73	7.64	7.58	6.94				
	W2	4.96	4.90	5.22	5.06	5.35	5.13	5.06	5.05	5.09				
	W3	4.75	5.37	4.63	4.52	4.38	4.95	4.51	4.82	4.74				
	Mean	5.75	6.11	5.85	5.70	5.61	5.71	5.94	5.92	5.82				
NDF (% DM)	W0	65.33	63.26	63.87	65.53	64.43	65.52	64.31	62.55	64.35	1.68	<0.001	0. 300	0.150
	W1	60.08	59.38	60.53	61.18	62.43	62.55	61.88	61.38	61.43				
	W2	62.71	68.44	67.64	73.56	69.96	70.01	72.01	70.59	69.37				
	W3	71.54	72.44	71.28	70.84	70.13	69.40	70.17	71.44	70.91				
	Mean	64.92	65.88	65.83	67.78	67.24	66.87	67.09	66.49	66.51				
ADF (% DM)	W0	40.12	39.38	39.09	40.39	40.68	40.49	40.09	40.65	40.11	1.72	<0.001	0.250	0.500
	W1	36.79	37.19	36.88	38.01	38.68	38.73	38.48	38.01	38.10				
	W2	36.88	42.45	42.23	47.42	44.24	44.52	45.69	45.55	43.62				
	W3	45.03	43.52	44.57	44.45	43.64	43.23	43.55	43.91	43.99				
	Mean	39.71	40.64	40.69	42.57	42.31	41.74	41.95	42.03	41.46				
HEM (% DM)	W0	25.21	23.88	24.77	25.14	23.75	25.02	24.23	21.91	24.24	0.65	<0.001	0.640	0.060
	W1	23.29	22.19	23.65	23.16	22.97	23.83	23.40	23.37	23.23				
	W2	25.83	25.98	25.41	26.14	25.72	25.50	26.32	25.03	25.74				
	W3	26.50	28.91	26.71	26.39	26.49	26.17	26.62	27.53	26.92				
	Mean	25.21	25.24	25.14	25.21	24.73	25.13	25.14	24.46	25.03				

DM, dry matter; CP, crude protein; NDF, neutral detergent fiber; ADF, acid detergent fiber; SEM, standard error of mean; FM, fresh weight; HEM, hemicellulose.

1) Treatments were 0-day wilting (W0), 1-day wilting (W^1)^, 2-day wilting (W^2)^, 3-day wilting (W3).

2) T, treatments; D, the days of ensiling; T×D, the interaction of treatments and ensiling days.

When p-value was smaller than 0.05, means were significantly different.

**Table 3:** Effect of different wilting periods on the dynamic change of RFV, TDN, and IVDMD of Italian ryegrass sampled at days of ensiling

Items	Treatments^1)^	Ensiling days (d)								Mean	SEM	p-value^2)^		
	
		1	2	3	5	10	20	30	45			T	D	T×D
IVDMD (% DM)	W0	61.55	64.53	62.95	60.77	58.79	62.91	61.95	61.94	61.92	1.31	0.14	<0.001	0.15
	W1	62.92	62.53	61.79	61.29	58.79	61.91	58.65	58.00	60.74				
	W2	59.87	60.07	62.45	59.50	58.91	61.11	57.97	64.67	60.57				
	W3	61.56	62.94	63.16	59.71	61.81	63.38	57.80	61.94	61.54				
	Mean	61.48	62.52	62.59	60.32	59.58	62.33	58.83	61.64	61.18				
TDN (%)	W0	57.20	57.79	58.02	56.99	56.76	56.91	57.23	56.79	57.21	1.01	<0.001	0.03	0.15
	W1	59.84	59.52	59.76	58.87	59.05	58.31	58.50	58.87	59.09				
	W2	59.76	58.69	55.54	54.77	53.95	53.73	52.81	52.91	55.27				
	W3	53.32	54.52	53.69	53.79	54.42	54.75	54.50	54.21	54.15				
	Mean	57.53	57.63	56.75	56.10	56.05	55.92	55.76	55.70	56.43				
RFV	W0	82.09	85.67	85.21	81.55	82.61	81.45	83.42	85.10	83.39	3.57	<0.001	0. 27	0.38
	W1	93.36	93.91	92.48	90.17	91.08	87.57	89.06	90.09	90.96				
	W2	89.26	77.04	77.65	67.80	72.38	72.04	68.90	70.39	74.43				
	W3	70.02	70.62	70.78	71.27	72.82	74.02	72.95	71.23	71.72				
	Mean	83.68	81.81	81.53	77.70	79.72	78.77	78.58	79.20	80.12				

RFV, relative feed value; TDN, total digestible nutrients; IVDMD, *in vitro* dry matter digestibility; SEM, standard error of mean; DM, dry matter.

1) Treatments were 0-day wilting (W0); 1-day wilting (W^1)^; 2-day wilting (W^2)^; 3-day wilting (W3).

2) T, treatments; D, the days of ensiling; T×D, the interaction of treatments and ensiling days.

When p-value was smaller than 0.05, means were significantly different.
